# PRESIDENTIAL SYMPOSIUM: EMBRACING ENGAGED TEACHING, RESEARCH, SERVICE, AND POLICY TO STRENGTHEN GERONTOLOGICAL EDUCATION

**DOI:** 10.1093/geroni/igae098.1390

**Published:** 2024-12-31

**Authors:** Christine Fruhauf, Tina Newsham

**Affiliations:** Chair; Discussant

## Abstract

It takes fortitude for GSA members to embrace engagement in their academic roles. Engagement is the opportunity for informing and enhancing research, teaching, and service, and policy by actively participating in activities that connect students with external partners and the community. In this AGHE Presidential Symposium, leading scholars whose grit and determination to embrace engagement will address the important role engagement has in uplifting gerontology and geriatrics education. Elinor Schoenfeld and colleagues will provide insights into their innovative, multidisciplinary approach to teaching students about community engagement, technology, and data security as it relates to supporting older adults living in their homes. Taylor Jansen will discuss incorporating Geographic Information Systems (GIS) Mapping into gerontology research and teaching both undergraduate and graduate students, as she works closely with community partners in several states across the U.S. to uplift their programming and policy work. José Carrión-Baralt will describe the process he and his colleagues embraced as they coordinated efforts across several universities and health professions to support older adults after hurricanes Irma and Maria hit Puerto Rico in 2017, through the development of a team called Brigadas Salubristas (Public Health Brigades). Shannon Jarrott will highlight ways to engage students in local, state, and federal aging policy through decades of engaged work addressing intergenerational programming. AGHE Chair Tina Newsham will serve as discussant, sharing how AGHE will continue to embrace fortitude and support educational efforts and opportunities through engaged teaching, research, service, and policy.

